# Supplementary material for: Dynalign II: common secondary structure prediction for RNA homologs with domain insertions
Source: Nucleic Acids Res. 2014 Nov 21;42(22):13939–48. doi: 10.1093/nar/gku1172 (PMC4267632; doi:10.1093/nar/gku1172)
Supplement: SUPPLEMENTARY DATA [file supp_gku1172_nar-02021-z-2014-File012.zip › manual/Text/oligoscreen.html]

RNAstructure Command Line Help: oligoscreen


|  |  |  |
| --- | --- | --- |
|  | RNAstructure Command Line Help  oligoscreen and oligoscreen-smp | - Contents - Index |
| oligoscreen is used to calculate folding free energy changes for formation of unimolecular, bimolecular, and duplex structures for a set of oligonucleotides. oligoscreen-smp is a parallel processing version for use on multi-core computers, built using OpenMP. USAGE: oligoscreen <list file> <report file> [options] OR: oligoscreen-smp <list file> <report file> [options] Required parameters: <list file> \| The name of a list file containing the oligos.; <report file> \| The name of a report file to which output will be written. The report is tab-delimited and provides columns for the input sequence (Sequence), the bimolecular folding free energy change for two identical oligonucleotides interacting (DGbimolecular), the folding free energy change for unimolecular self-structure (DGunimolecular), the folding free energy change for duplex formation with the complementary sequence (DGduplex), the cost of opening the two base pairs at the 5' end of the oligonucleotide in a duplex with the complementary sequence (DG2BPat5'), and the cost of opening the two base pairs at the 3' end of the oligonucleotide in a duplex with the complementary sequence (DG2BPat3'). Folding free energy changes are in kcal/mol. Options which don't require added values: -d, -D, --DNA \| Specify that the sequence is DNA, and DNA parameters are to be used. Default is to use RNA parameters.; -h, -H, --help \| Display the usage details message. Options which require added values: -t, -T, --temperature \| Specify the temperature at which calculation takes place in Kelvin. Default is 310.15 K, which is 37 degrees C. Notes for smp: oligoscreen-smp, by default, will use all available compute cores for processing. The number of cores used can be controlled by setting the OMP\_NUM\_THREADS environment variable. References: 1. Reuter, J.S. and Mathews, D.H. "RNAstructure: software for RNA secondary structure prediction and analysis." *BMC Bioinformatics*, 11:129. (2010). 2. Matveeva, O.V., Mathews, D.H., Tsodikov, A.D., Shabalina, S.A., Gesteland, R.F., Atkins, J.F. and Freier, S.M. "Thermodynamic criteria for high hit rate antisense oligonucleotide design." *Nucleic Acids Res.*, 31:4989-4994.(2003). | | |
| Visit The Mathews Lab RNAstructure Page for updates and latest information. | | |
